# Malnutrition Risk in Older Adults: Evaluating the Diagnostic Relevance of Serum Biomarkers: SIRT-1, CCK-8, Melatonin, and Total Antioxidant Capacity (TAC)

**DOI:** 10.3390/nu17040726

**Published:** 2025-02-18

**Authors:** Karolina Kujawowicz, Iwona Mirończuk-Chodakowska, Monika Cyuńczyk, Anna Maria Witkowska

**Affiliations:** Department of Food Biotechnology, Medical University of Białystok, ul. Szpitalna 37, 15-285 Białystok, Poland; iwona.mironczuk-chodakowska@umb.edu.pl (I.M.-C.); monika.cyunczyk@umb.edu.pl (M.C.); anna.witkowska@umb.edu.pl (A.M.W.)

**Keywords:** malnutrition, risk, elderly, serum, SIRT-1, CCK-8, melatonin, total antioxidant capacity

## Abstract

**Background/Objectives:** Addressing the risk of malnutrition at an early stage is crucial to preventing its development, which can have a detrimental impact on physical and mental health status. This study investigates the potential role of biochemical biomarkers such as sirtuin 1 (SIRT-1), melatonin, cholecystokinin-8 (CCK-8), and total antioxidant capacity (TAC) in identifying the risk of malnutrition. **Methods:** This cross-sectional study assessed malnutrition risk in 153 community-dwelling older adults using the Mini Nutritional Assessment (MNA). Serum levels of SIRT-1, melatonin, and CCK-8 were analyzed with enzyme-linked immunosorbent assay (ELISA), and total antioxidant capacity (TAC) was measured using the ferric reducing ability of plasma (FRAP) method. **Results:** Serum levels of TAC and CCK-8 were significantly positively correlated with grip strength and visceral adipose tissue, with TAC levels also showing associations with appendicular skeletal muscle mass index (ASMI), total body water, total energy expenditure, fat-free mass index, and fat mass index (*p* < 0.001). CCK-8 emerged as a strong predictor of malnutrition risk (AUC = 0.58 in females, AUC = 0.64 in males), whereas SIRT-1 (AUC = 0.57 for both sexes), melatonin (AUC = 0.46 for females, AUC = 0.51 for males), and TAC (AUC = 0.42 for females, AUC = 0.54 for males) exhibited weaker predictive abilities. A multivariate model incorporating CCK-8 demonstrated excellent predictive accuracy (AUC = 0.84, 95% CI: 0.77–0.90) and indicated a potential association between elevated CCK-8 levels and a higher risk of malnutrition. **Conclusions:** In conclusion, this study highlights the effectiveness of a multi-parameter model incorporating CCK-8 as a reliable approach for assessing malnutrition risk in older adults, offering a comprehensive evaluation of the condition. However, further research is needed to confirm its applicability and accuracy in diverse elderly populations and clinical settings.

## 1. Introduction

According to the European Society of Clinical Nutrition and Metabolism (ESPEN), malnutrition encompasses a range of conditions, including disease-related cachexia and sarcopenia. It is defined based on Body Mass Index (BMI) thresholds, unintentional weight loss, or fat-free mass index [1]. Malnutrition in the elderly represents a significant health issue that can result in a range of adverse outcomes [2], including muscle weakness [3], weight loss [4,5], compromised immune function [6], and an elevated risk of chronic disease and mortality [7,8,9]. The condition is influenced by a combination of physiological and psychosocial factors. Physiological changes include a reduction in appetite and nutrient absorption. Psychosocial factors include feelings of loneliness and depression [10,11,12]. Various tools are available for assessing the risk of malnutrition; however, the Mini Nutritional Assessment (MNA) is specifically recommended for older adults [13].

Existing research on malnutrition in the elderly predominantly focuses on clinical indicators such as albumin levels and BMI [14,15]. However, there is a paucity of research on more subtle factors that may be crucial for understanding the risk of malnutrition and its health consequences. These include oxidative processes [16,17] associated with inflammation and circadian rhythms [18]. Therefore, further research is required to identify biomarkers [19] that can be used to diagnose malnutrition, develop interventions to improve the quality of life of older people, and reduce the risk of diseases associated with nutritional deficiencies [20,21]. The development of effective diagnostic tools for malnutrition represents a pivotal objective within the domain of geriatric research [22]. This study presents a review of the existing literature on malnutrition, with a particular focus on several laboratory parameters and their potential role in assessing malnutrition in older people.

SIRT-1, a member of the sirtuin family, is an NAD-dependent enzyme that deacetylates histones and transcription factors that regulate circadian rhythms and energy metabolism [23]. SIRT-1 plays a pivotal role in the regulation of energy metabolism, aging processes, inflammation, apoptosis, and stress responses [24], primarily through the deacetylation of crucial regulators such as p53, FOXO, and PGC-1α [25,26]. Consequently, it may serve as a potential indicator of the body’s adaptive capacity to undergo changes in response to aging. A potential link between malnutrition and SIRT-1 has been previously described [27].

The hormone melatonin is responsible for regulating circadian rhythms [28], and it has been demonstrated that this may affect metabolic processes, particularly in older adults [29]. It is well established that sleep and other biological rhythms are subject to disturbance in the elderly [30,31]. Given its antioxidant and anti-inflammatory effects, melatonin may play a role in the context of malnutrition in the elderly. Conversely, tryptophan (Trp) deficiency, which can occur in malnutrition, has been shown to negatively affect melatonin synthesis, which can disrupt bodily functions related to circadian rhythm regulation and homeostasis [32].

Cholecystokinin octapeptide (CCK-8) is a peptide that plays a role in the brain–gut axis. It is present in both the gastrointestinal tract, where it affects gallbladder contraction and regulates pancreatic secretion, and in the central nervous system, where it acts as a neurotransmitter or neuromodulator [33,34,35]. The levels of CCK-8 in the bloodstream are known to rise in response to the intake of food, indicating that the peptide may play a role in regulating satiety levels and influencing overall dietary intake. Studies have documented the ability of CCK-8 to exert a suppressive influence on food consumption, particularly in the context of sham feeding [36]. The capacity of CCK-8 to regulate appetite and impact nutritional status is a topic of particular interest in the geriatric field, given that nutritional deficiencies are a common concern in this age group [37].

**Figure 1:**
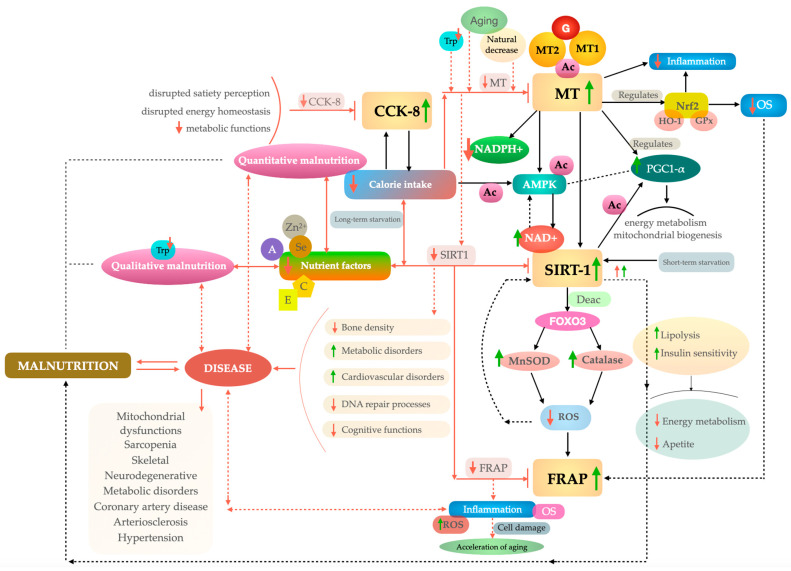
Hypothetical model, based on a literature review, illustrating potential associations of SIRT-1, melatonin, CCK-8, and FRAP with malnutrition in older adults. Notes: CCK-8—cholecystokinin-8; Trp—tryptophan; Zn^2+^—zinc; A—vitamin A; Se—selenium; C—vitamin C; E—vitamin E; MT—melatonin; G—G-protein; MT1—melatonin receptor 1; MT2—melatonin receptor 2; Ac—activation; Nrf2—nuclear factor erythroid 2-related factor 2; FOXO3—forkhead box O3; MnSOD—Manganese superoxide dismutase; ROS—reactive oxygen species; FRAP—fluorescence recovery after photobleaching; SIRT-1—sirtuin 1; GPx—glutathione peroxidase; HO-1—haemoxygenase-1; OS—oxidative stress; AMPK—AMP-activated protein kinase; PGC-1α—peroxisome proliferator-activated receptor-gamma coactivator; NADPH+—nicotinamide adenine dinucleotide phosphate; NAD+—nicotinamide adenine nucleotide; Deac—deacetylation. Green upwards arrows indicate increase and red downwards arrows indicate decrease.

In light of the findings of studies on malnutrition, an assessment of the total antioxidant activity (TAC) of the body may prove to be a promising avenue of enquiry. A widely used methodology in this regard is the ferric reducing ability of plasma (FRAP) assay [38,39], which is employed for evaluating antioxidant capacity and its reduction potential [40]. Malnutrition in the elderly has been demonstrated to increase oxidative stress, which can intensify the likelihood of health complications such as cellular damage and cognitive impairment [41,42]. Consequently, FRAP, as a method for evaluating antioxidant capacity, offers insights into prospective deficiencies in antioxidant defense, which is vital in assessing the influence of nutritional status on health [43]. Potential correlations between the aforementioned parameters are illustrated in Figure 1.

Figure 1 illustrates the multifaceted interplay between melatonin, SIRT-1, CCK-8, and FRAP antioxidant activity within the context of malnutrition among elderly individuals. G-protein-coupled melatonin receptors MT1 and MT2 bind with melatonin, leading to an increase in melatonin levels [44]. A reduction in tryptophan concentration and the process of physiological aging are associated with a decline in melatonin production in human serum [45]. Melatonin, a potent antioxidant [46,47,48], exerts its effects by mitigating oxidative stress [49,50] and enhancing the NAD+/NADH ratio, which ultimately activates the SIRT-1 [51]. An increase in SIRT-1 activity results in the deacetylation of proteins such as FOXO3, which in turn activates antioxidant enzymes, including MnSOD and catalase, thereby protecting against oxidative damage [52]. This results in a reduction in ROS (reactive oxygen species) and an increase in FRAP. Excessive increases in SIRT-1 may contribute to short-term starvation [53,54], as well as increased lipolysis [55] and insulin sensitivity [56,57], resulting in a decrease in energy metabolism and appetite. This may indirectly lead to the manifestation of malnutrition.

Furthermore, melatonin modulates the Nrf2 (nuclear factor erythroid 2-related factor 2) pathway, which regulates the expression of genes encoding antioxidant enzymes, including glutathione peroxidase (GPx) and haemoxygenase-1 (HO-1) [58,59]. This, in turn, contributes to a reduction in inflammation and oxidative stress (OS) [60,61], resulting in an indirect increase in FRAP [62]. An increase in SIRT-1 leads to an enhancement of AMPK activity [63]. The SIRT-1, AMPK, and PGC-1α (peroxisome proliferator-activated receptor-gamma coactivator) proteins constitute a complex that regulate mitochondrial biogenesis and cellular energy metabolism [64]. Caloric restriction activates AMPK and SIRT-1, thereby increasing ATP production through fat oxidation [65]. Melatonin affects the regulation of the transcriptional coactivator PGC-1α, which, in turn, is activated by SIRT-1 [66].

PGC-1α regulates mitochondrial biogenesis and energy metabolism [67]. Activation of this pathway enhances mitochondrial function and protect cells from oxidative stress, thereby promoting the effects of SIRT-1 [68].

A reduction in CCK-8 may result in a dysregulation of appetite [69] and metabolic processes. Conversely, an increase in CCK-8 may lead to a decrease in appetite and caloric intake. These effects may be reciprocal [70,71,72].

Caloric restriction may both increase and decrease SIRT-1 activity through the activation of AMPK [73]. In general, AMPK activation results in elevated levels of SIRT-1 activity [74,75]. Excess fat or carbohydrate deficiency leads to a decrease in AMPK activity, which can reduce SIRT-1 activity [76,77].

A reduction in key nutrient factors—such as antioxidants including selenium (Se) [78,79], zinc (Zn^2+^) [80,81,82], vitamin C (notably influencing SIRT-1 expression: the protective effects of vitamin C against oxidative stress are linked to the SIRT-1 signaling pathway due to SIRT-1’s regulatory impact on p53 and FOXO3) [83], and vitamin E [84,85]—may contribute to a decline in SIRT-1 activity [86].

A reduction in SIRT-1 activity may contribute to an increased risk of developing diseases [87,88] that are associated with malnutrition [89,90]. Such conditions include neurodegenerative disease [91,92,93] associated with malnutrition [94,95]. Furthermore, there is an established link between skeletal diseases such as osteoporosis and malnutrition [96,97,98,99]. Metabolic disorders [100] and mitochondrial dysfunctions [101] have been linked to the development of sarcopenia [102]. Arterioslerosis [103], coronary artery disease [104] and hypertension [105,106] are also closely associated with malnutrition [107,108,109,110,111].

Low TAC levels in plasma indicate a reduction in antioxidant capacity, which consequently increases oxidative stress [112]. Oxidative stress and inflammation are associated with an increase in reactive oxygen species (ROS) [113], which in turn contributes to disease [114]. Furthermore, inflammation can be induced by or exacerbate oxidative stress, leading to cell destruction and accelerating the aging process [115]. Scientific evidence suggests that malnutrition, inflammation, and oxidative stress are inter-related [116].

The proposed scheme demonstrates how diminished melatonin levels, reduced SIRT-1 activity, and aberrant CCK-8 regulation can precipitate a multitude of deleterious alterations, including inflammation and oxidative stress. These changes may subsequently predispose individuals to the development of malnutrition-related illnesses or directly result in the onset of malnutrition.

Early identification of malnutrition risk is crucial to prevent its progression, as it can severely impact physical, mental, and overall health in the elderly population. This study examines several key biochemical markers, including SIRT-1, melatonin, cholecystokinin-8 (CCK-8), and total antioxidant capacity (TAC), which may play a vital role in metabolic regulation and reflect the nutritional status of the elderly population. By comparing these biomarkers with clinical nutritional assessments, this research study aims to determine their utility as predictors of malnutrition risk.

## 2. Materials and Methods

### 2.1. Study Group

A total of 153 individuals (77% females) aged 60 to 82 years participated in this cross-sectional study, thus exceeding the minimum required sample size that had been established through power analysis. The analysis determined that 52 participants per group (at risk and not at risk of malnutrition) were necessary to detect a mean effect size (d = 0.6) with a power of 0.8 and alpha of 0.05 in a two-sided *t*-test. To ensure reliability, the sample size was increased by 15% to account for potential non-parametric tests and rounded up, ensuring balanced group recruitment and applicability of results to a broader population.

This study builds upon and expands our previous research. It was conducted at community centers, educational institutions for the elderly, and support organizations, as previously described [117]. Individuals were eligible to participate in this study if they met the following criteria: age, absence of cancer, willingness to engage in this study at any stage, BMI above 18.5 kg/m^2^, ambulatory and able to walk independently, absence of fever, stability of chronic diseases, and blood serum meeting laboratory requirements. One subject was excluded from the present study compared to the previous analysis, as their serum sample did not meet the necessary criteria for laboratory analysis. The exclusion criteria included the following conditions: dementia, active infectious disease (e.g., hepatitis B or C, HIV infection), current use of antibiotics or anti-inflammatory drugs, dysphagia or severe gastrointestinal disorders, parenteral or enteral nutrition, inflammatory bowel conditions (e.g., diagnosed celiac disease, short bowel syndrome, pancreatic insufficiency), end-stage liver and/or kidney failure, acute myocardial infarction within the past 30 days, or active malignant tumor within the past five years. A total of 154 subjects were initially enrolled in the study. However, after applying the inclusion and exclusion criteria, 153 were ultimately deemed eligible for participation. In both the preceding and present study, the aggregate number of subjects categorized as being at risk of malnutrition was 58. Moreover, the median age in both studies remained constant at 69 years. In addition, no discrepancies were detected between the studies with regard to marital status, lifestyle, or educational attainment. Furthermore, participants maintained their usual diet. The detailed characteristics of the previous study participants are described in [117]. In brief, the at-risk group and the well-nourished group did not differ in terms of chronic diseases, except for higher rates of depression (Geriatric Depression Scale) and osteoporosis (based on medical history) in the at-risk group. Selective serotonin reuptake inhibitors (SSRIs) and monoamine oxidase inhibitors (MAOIs) were significantly more frequently used in the group at risk of malnutrition. With regard to anthropometric measurements and physical ability, no statistically significant differences were observed between the groups analyzed in the previous study and those in the current study.

Venous blood was collected from the elbow vein into a tube containing a clotting activator. Additionally, venous blood was obtained from participants in the morning (between 8:00 and 9:00 a.m.) following an overnight fast. Within an hour of collection, the blood was centrifuged at 3000× *g* for 10 min to obtain serum. After centrifugation, the serum was expeditiously transferred into a fresh, sterile tube and stored at −80 °C in accordance with manufacturer guidelines until analysis. In cases where hemolysis occurred, the serum sample was excluded from this study.

This study was conducted in accordance with ethical guidelines designed to ensure the protection of participants’ autonomy, privacy, and dignity. These measures were implemented with the dual objective of obtaining reliable data and ensuring the safety and comfort of all those involved. This study was approved by the Bioethics Committee of the Medical University of Bialystok (approval number APK.002.421.2021) and was conducted in accordance with the Declaration of Helsinki.

### 2.2. Functional Assessment

In order to investigate potential relationships with biochemical parameters, this study employed a series of methods designed to assess the functional status of older people as previously described [117]. These included the administration of questionnaires such as the Mini Nutritional Assessment (MNA) [118], the Geriatric Depression Scale (GDS) [119], the Simplified Nutritional Appetite Questionnaire (SNAQ), and Council on Nutrition Appetite Questionnaire (CNAQ) [120]. Furthermore, this study employed measuring handgrip strength, gait speed, and body composition via bioelectrical impedance analysis (BIA) using the SECA mBCA 525 analyzer, seca gmbh & co.kg, Hamburg, Germany.

As the Mini Nutritional Assessment (MNA) is crucial for the classification of older people with and without malnutrition risk, the classification of subjects is described in detail. The Mini Nutritional Assessment—Long Form (MNA-LF) was utilized in this study to evaluate the risk of malnutrition [121]. This validated tool comprises 18 items that assess anthropometric data, dietary habits, appetite, health, and functional capacity. Scores on this scale range from 0 to 30, with classifications as follows: malnourished (<17), at risk of malnutrition (17–23.5), or nutritionally adequate (≥24). Participants scoring 17–23.5 were categorized as at risk of malnutrition, while those with scores ≥24 were considered to have satisfactory nutritional status.

### 2.3. Assessment of Frailty

Frailty is clinically defined as a state in which older individuals have diminished capacity to cope with everyday or acute stressors resulting from increased vulnerability due to age-related declines in physiological reserve and function across multiple organ systems [122].

The condition is common among older adults and varies by demographic group, highlighting the need for accurate assessment and intervention. In the present study, the diagnosis of frailty syndrome was based on the presence of at least three of the five factors outlined by Fried [123]:

1. A subjective experience of diminished strength or fatigue;
2. Feeling of fatigue;
3. Weight loss (at least 5 kg in a year);
4. Slowing gait speed;
5. Low physical activity.

### 2.4. Determination of SIRT-1, Melatonin, and CCK-8 in Serum

SIRT-1, melatonin, and CCK-8 were determined in serum by the ELISA technique using commercial kits. SIRT-1 was determined using ELISA Human NAD-dependent deacetylase sirtuin 1 (SIRT1/SIR2L1), Cat. No. CSB-E15058h, Cusabio, Houston, TX, USA. The detection range was 0.156–10 ng/mL and the sensitivity of the assay is 0.039 ng/mL. The precision was SIRT-1: >8% (intra-assay) and >10% (inter-assay).

Melatonin concentration was assessed with the Melatonin ELISA Kit, Cat. No. EU0199, Wuhan, China. The detection range was 7.813–500 pg/mL, and the sensitivity of the assay was 4.688 pg/mL. Precision was 5.41% (intra-assay) and 4.55% (inter-assay).

Cholecystokinin-8 levels were measured using the Human CCK-8 (Cholecystokinin 8) ELISA Kit, Cat. No. EH2771, Wuhan, China. The detection range was 16.625–1000 pg/mL and the sensitivity of the assay was 9.375 pg/mL. Precision was 5.13% (intra-assay) and 5.45% (inter-assay).

All assay procedures were carried out following the manufacturer’s recommendations provided with each kit.

Absorbance was measured for each sample using an ELISA plate reader (Rayto, RT-6100 Microplate Reader, Rayto Life and Analytical Sciences Co., Ltd., Shenzhen, China) at a wavelength of 450 nm. Standards and SIRT-1, melatonin, and CCK-8 were prepared and measured in duplicate. The concentration of SIRT-1, melatonin, and CCK-8 in the samples was determined by comparing the optical density of the samples with the standard curve.

### 2.5. Total Antioxidant Capacity (TAC)

TAC in serum samples was evaluated using the ferric reducing ability of plasma (FRAP) method according to Benzie and Strain [39].

This method measures the ability of a sample to reduce Fe^3+^ ions to Fe^2+^ ions. The reduction of Fe^3+^ to Fe^2+^ forms a complex with TPTZ (2,4,6-tris(2-pyridyl)-1,3,5-triazine), producing an intense blue color with a maximum absorbance at 593 nm. The antioxidant capacity is determined by comparing the change in absorbance (ΔA) of the sample to that of a standard Fe^2+^ solution. The FRAP unit represents the amount of Fe^3+^ reduced to Fe^2+^ per mole [124]. In this study, 1.5 mL of freshly prepared FRAP reagent was heated to 37 °C, and the blank was read at 593 nm. Subsequently, 100 μL of the test sample and 200 μL of H_2_O were added, mixed, and incubated in a water bath at 37 °C for 4 min. Absorbance (ΔA) was measured at 593 nm using a UV-1800 spectrophotometer (Shimadzu Corporation, Kyoto, Japan). Results were calculated using the calibration curve. Samples were measured in duplicate.

### 2.6. Statistical Analysis

Continuous variables were presented as medians (Mdn) and interquartile ranges (IQR), while categorical variables were expressed as frequencies (n) and percentages. To compare continuous variables, the Wilcoxon rank-sum test was applied, while Pearson’s chi-square or Fisher’s exact test, depending on sample size and expected frequencies, was used for categorical data.

For the analysis of the optimal cut-off points for biochemical and molecular biomarkers in relation to the risk of malnutrition, the cut-off points were determined using the maximize metric method, which identifies the threshold that maximizes the sum of sensitivity and specificity. The analysis was stratified by sex to account for potential differences between male and female subgroups.

The model’s performance was evaluated using various metrics, including sensitivity, specificity, accuracy (ACC), and the area under the ROC curve (AUC). Sensitivity and specificity were calculated for each subgroup (male and female), and the optimal cut-off points for the studied parameters were determined separately for each sex.

The association between two numerical variables, without assuming normality, was assessed using the Spearman correlation method. The strength and direction of the correlation were quantified by Spearman’s Rho coefficient. The statistical significance of the correlation was determined using an asymptotic approximation of the *t*-test statistic.

The multivariate effects of predictors on the risk of malnutrition, with results derived from the Mini Nutritional Assessment (MNA) questionnaire. A stepwise selection algorithm guided by the Akaike Information Criterion (AIC) refined the model. Predictor effects were expressed as odds ratios (ORs), while Tjur’s R^2^ assessed model explanatory power. The Hosmer–Lemeshow test evaluated model fit, and a 10-fold cross-validation procedure assessed performance, focusing on accuracy and Kappa statistics. Variance Inflation Factor (VIF) identified multicollinearity, with values above 10 indicating potential concern. The AUC of the ROC curve quantified discriminative ability, with confidence intervals estimated using the DeLong test.

A power analysis ensured adequate sample sizes for statistical tests at α = 0.05 and a power of 80% (1 − β = 0.80). For Spearman’s correlation, a minimum of 122 participants was required to detect a coefficient of 0.25. For group comparisons, the two-sample *t*-test required 76 participants (38 per group), adjusted to 88 for non-parametric tests. For chi-squared tests of independence with an effect size of w = 0.30, a minimum of 107 participants was calculated.

A significance level of *p* < 0.05 was set. All analyses were conducted using R Statistical Software (version 4.3.3), with relevant packages including pwr, sjPlot, performance, report, correlation, pROC, gtsummary, cutpointr, gofcat, MASS, and dplyr, on Windows 11 Pro (64-bit).

## 3. Results

The study investigated four assayed serum parameters—sirtuin1 (SIRT-1), melatonin (MT), cholecystokinin-8 (CCK-8), and total antioxidant activity (TAC). Table 1 presents the distribution of the studied parameters for the overall sample, as well as for the subsamples stratified according to the risk of malnutrition as defined by the Mini-Nutritional Assessment (MNA) tool.

No statistically significant difference was observed between the two groups regarding serum SIRT-1 (*p* = 0.265). Similarly, no significant difference was observed in CCK-8 levels between the malnutrition risk group and the non-malnutrition risk group (*p* = 0.114).

Melatonin serum concentrations in both groups were found to be similar (*p* = 0.362), and no significant association was identified between these levels and the risk of malnutrition in this sample.

Furthermore, no significant difference was observed between the two groups with respect to SIRT-1 (*p* = 0.265), suggesting that it may not be strongly associated with nutritional risk in this cohort. Similarly, CCK-8 demonstrated no statistically significant difference between the group at risk of malnutrition and the control group (*p* = 0.114). However, a trend toward higher levels was observed in individuals at risk of malnutrition.

TAC showed only a trend toward lower levels in the group at risk of malnutrition compared to the group without risk (*p* = 0.090).

### 3.1. Identification of Optimal Thresholds for SIRT-1, Cholecystokinin-8, Melatonin, and TAC in Assessing Malnutrition Risk

In assessing the predictive value of SIRT-1, cholecystokinin-8, melatonin, and TAC for the risk of malnutrition, optimal cut-off points were established for both male and female participants. These biomarkers include SIRT-1, cholecystokinin-8, melatonin, and TAC. Performance metrics such as accuracy, sensitivity, specificity, and AUC were calculated for each sex-specific cut-off to evaluate their effectiveness in distinguishing between individuals at risk of malnutrition. Table 2 provides a summary of the optimal cut-off points and corresponding performance metrics for each biomarker with the risk of malnutrition.

SIRT-1 showed a moderate predictive ability in both sexes, with an AUC = 0.57 in males and females. In females, sensitivity was relatively high (0.77), but the low specificity (0.39) raises concerns about false positives. Similarly, in males, SIRT-1 exhibited a sensitivity of 0.80 but a low specificity (0.44), indicating a tendency to misclassify well-nourished individuals as malnourished.

Cholecystokinin-8 (CCK-8) demonstrated slightly better performance compared to other biomarkers. In females, it achieved an AUC = 0.58, with moderate accuracy (0.61) and specificity (0.69), though sensitivity was modest (0.50). In males, CCK-8 performed better, with an AUC = 0.64, high sensitivity (0.90), and moderate specificity (0.44). The high sensitivity in males suggests effectiveness in identifying malnutrition, although the lower specificity may lead to some false positives. Clinically, CCK-8 shows promise, particularly in males, as a biomarker for early malnutrition detection.

Melatonin showed the poorest performance among the biomarkers evaluated. In females, it showed an AUC = 0.46, with very high sensitivity (0.96) but extremely low specificity (0.10). This indicates that while highly sensitive to malnutrition, it misclassifies nearly all well-nourished individuals, rendering it unsuitable for clinical use as a standalone marker. Similarly, in males, melatonin had an AUC = 0.51, with perfect sensitivity (1.00) but low specificity (0.20).

TAC demonstrated limited discriminative power in both females and males. In females, TAC showed a low AUC = 0.42, indicating poor overall predictive value. Although sensitivity was moderate (0.67), the low specificity (0.34) suggests frequent misclassification of well-nourished individuals as malnourished, resulting in a high false-positive rate. In males, TAC exhibited better specificity (0.96), making it more effective for ruling out malnutrition. However, the low sensitivity (0.30) and moderate AUC (0.54) limit its reliability as a diagnostic tool for detecting malnutrition in men. Clinically, TAC may serve better as a confirmatory test in males rather than a primary screening tool.

Table A1, included in the Appendix A, presents non-stratified cut-off points and performance metrics for the entire population. By synthesizing data without sex-specific divisions, it complements the main findings and ensures broader applicability in diverse research and clinical contexts. This addition bridges the gap between the precision of sex-specific analyses with the universal relevance required for general use, enhancing this study’s overall utility.

### 3.2. Estimation of Correlations Among Biochemical and Molecular Biomarkers

It is crucial to understand the correlations between SIRT-1, CCK-8, melatonin, and TAC in order to explore their roles in metabolic regulation, oxidative stress, and appetite control. Table A2 examines these biomarkers to identify potential pathways involved in malnutrition and related health conditions. The correlation analysis between the biochemical and molecular biomarkers showed no statistically significant associations after adjusting for multiple comparisons, limiting their combined diagnostic utility in malnutrition risk. TAC exhibited weak and non-significant correlations with melatonin (Rho = −0.05) and CCK-8 (Rho = 0.10), suggesting TAC’s independence from these hormonal biomarkers in oxidative stress and metabolic regulation. SIRT-1 showed a modest, non-significant positive correlation with melatonin (Rho = 0.18, padj = 0.172), hinting at a potential link between circadian and metabolic regulation. However, its correlations with TAC (Rho = 0.02) and CCK-8 (Rho = 0.12) were weak, further reducing clinical relevance. CCK-8 demonstrated no significant correlations with melatonin (Rho = 0.06), TAC (Rho = 0.10), or SIRT-1 (Rho = 0.12), indicating a limited interaction with these markers in appetite regulation or malnutrition risk (refer to Table A1 in the Appendix A for full details).

### 3.3. Evaluation of Assayed Serum Biomarkers Across Smoking Status and Clinical Comorbidities

Table 3 presents the distribution of key biochemical and molecular biomarkers—SIRT-1, CCK-8, melatonin, and TAC—stratified by various clinical comorbidities, including smoking status, hypertension, diabetes, cardiovascular disease (CVD), hypothyroidism, hyperlipidemia, and obesity. These analyses aim to explore potential differences in biomarker levels between individuals with and without specific comorbidities, providing insights into how these health conditions may influence or be associated with changes in oxidative stress, metabolic regulation, and hormonal markers.

There were no significant differences in individuals with a history of smoking in the levels of SIRT-1, CCK-8, melatonin or TAC when compared to non-smokers. Despite the absence of statistically significant differences between the groups, caution should be exercised when interpreting the results. This is due to the unequal group sizes, which may have affected our ability to detect effects.

Hypertensive individuals, on the other hand, showed a significant elevation in TAC (*p* = 0.004), indicating increased oxidative stress compared to those without hypertension. Although SIRT-1 levels trended lower in hypertensive participants, this difference was not statistically significant (*p* = 0.119). CCK-8 levels were marginally higher in hypertensive older adults (*p* = 0.060. Melatonin levels did not differ significantly between hypertensive and non-hypertensive individuals.

In individuals with diabetes, there were no significant differences observed in SIRT-1, CCK-8, melatonin, or TAC compared to non-diabetic individuals.

According to the results in Table 2, SIRT-1 levels were lower in older adults with cardiovascular disease, although this difference did not reach statistical significance (*p* = 0.416). Higher levels of CCK-8 were observed in individuals with cardiovascular disease (*p* = 0.067), but this result was not statistically significant. Melatonin levels were not significantly different between the two groups. However, older adults with cardiovascular disease exhibited significantly elevated TAC levels (*p* = 0.030), indicating greater oxidative stress compared to those without cardiovascular disease.

No significant differences were observed in SIRT-1, CCK-8, or melatonin levels in elderly participants with hypothyroidism. In individuals with hypothyroidism, there was a statistically significant reduction in TAC levels (*p* = 0.047), suggesting lower oxidative stress in these group.

Individuals with hyperlipidemia showed no significant difference in SIRT-1 or TAC levels compared to those without hyperlipidemia. However, CCK-8 levels were significantly higher in individuals with hyperlipidemia (*p* = 0.019), which could reflect alterations in appetite regulation and lipid metabolism associated with dyslipidemia. Melatonin levels did not differ significantly between hyperlipidemic and non-hyperlipidemic individuals.

### 3.4. Correlations Between Assayed Biomarkers and Functional and Body Composition Assessments

The relationship between biochemical and molecular biomarkers and various clinical assessments can provide valuable insights into the underlying mechanisms linking metabolic and physiological health. By examining the correlations of markers such as SIRT-1, CCK-8, melatonin, and TAC with functional, nutritional, and body composition parameters, it is possible to identify potential pathways that contribute to the development of frailty syndrome, probable sarcopenia, and other age-related conditions. These correlations facilitate the comprehension of the manner in which oxidative stress, metabolic regulation, and hormonal balance are reflected in physical performance measures, including grip strength and body composition indices such as ASMI and FFMI, as well as energy expenditure and risk factors such as depression and frailty syndrome. Table 4 presents the results of these correlations, offering a more nuanced understanding of the interactions between biochemical markers and clinical outcomes.

SIRT-1 showed weaker correlations overall. Although SIRT-1 had a modest positive correlation with loss of appetite (Rho = 0.14, *p* = 0.080), this association did not reach statistical significance. SIRT-1 was negatively correlated with fat mass index (FMI) (Rho = −0.16, *p* = 0.054), but this result was also marginally non-significant. SIRT-1 did not show strong relationships with other functional or body composition parameters in this cohort.

CCK-8 displayed a positive correlation with grip strength (Rho = 0.16, *p* = 0.044). Additionally, CCK-8 was significantly correlated with visceral adipose tissue (Rho = 0.22, *p* = 0.007). Although CCK-8 showed some positive trends with other body composition measures, including ASMI and FFMI, these associations were not statistically significant.

Melatonin did not demonstrate any significant correlations with the majority of the clinical assessments. Melatonin’s most notable relationship was a marginally non-significant positive correlation with resting energy expenditure (Rho = 0.14, *p* = 0.075). However, no strong associations were observed between melatonin and muscle strength, frailty, or body composition measures in this sample.

TAC exhibited several significant positive correlations. Notably, TAC was positively associated with grip strength (Rho = 0.22, *p* = 0.006). Furthermore, TAC demonstrated strong correlations with body composition parameters, including ASMI (Rho = 0.38, *p* < 0.001), TBW (Rho = 0.35, *p* < 0.001), TEE (Rho = 0.36, *p* < 0.001), REE (Rho = 0.39, *p* < 0.001), FFMI (Rho = 0.35, *p* < 0.001), and VAT (Rho = 0.41, *p* < 0.001).

No significant differences were observed between the current study and previous ones regarding the distribution of clinical, functional, nutritional, and body composition assessments, both for the overall sample and stratified by malnutrition risk. However, a novel parameter included in the current study is the assessment of frailty syndrome risk. The prevalence of frailty syndrome was markedly higher in the risk malnutrition group (60.34%) than in the group without the risk of malnutrition (17.89%) (*p* < 0.001), indicating a robust correlation between nutritional risk and increased frailty. The results effects are presented in Table A3 in the Appendix A.

### 3.5. Identification of Determinants of Malnutrition Risk Among Older People Using a Multivariate Analysis

#### 3.5.1. Model Development and Predictor Selection Procedure

Initially, a logistic regression model was developed to examine the impact of 37 clinical predictors—sex, age, BMI, education, smoker, hypertension, diabetes, cardiovascular diseases, hypothyroidism, hyperlipidemia, polypharmacy, obesity, severe illnesses in the last year, hospitalizations in the last year, statin therapy, antihypertensive medication, selective serotonin reuptake inhibitors, thyroid hormone replacement therapy, diuretic medications, antidiabetic medications, grip strength, risk of frailty syndrome, depression risk (GDS), loss of appetite (according to SNAQ and CNAQ), probable sarcopenia (according to SARC-F), appendicular skeletal muscle mass index level (ASMI), total body water (TBW), total energy expenditure (TEE), resting energy expenditure (REE), fat-free mass index (FFMI), fat mass index (FMI), phase angle (PA), SIRT-1, CCK-8, melatonin, and TAC—on the occurrence risk of malnutrition. During the model fitting process, variables such as BMI, REE, TBW, and FFMI were sequentially excluded, one by one, due to multicollinearity. This iterative approach was necessary to ensure the stability and reliability of the final model by eliminating predictors that exhibited high intercorrelation, which could otherwise distort the interpretation of the regression coefficients.

Through the application of a stepwise algorithm with backward elimination, the number of model predictors was reduced from 33 to 7, leading to a decrease in the Akaike Information Criterion (AIC) from 204.74 to 164.70. Interestingly, among the four parameters of prior interest—SIRT-1, CCK-8, melatonin, and TAC—only CCK-8 was identified as being significantly associated with the response variable.

This reduction in AIC reflects a significant improvement in the model’s overall fit and parsimony, indicating that the final model with smoking, SSRI medication, frailty syndrome, loss of appetite (according to SNAQ and CNAQ), ASMI, VAT, and CCK-8 predictors offers a more efficient and accurate representation of the data while mitigating the risk of overfitting.

#### 3.5.2. Hosmer–Lemeshow Test Results

Overall, the results of the Hosmer–Lemeshow test χ^2^ (8) = 7.695, *p* = 0.464 suggest that the logistic regression model does not exhibit a significant lack of fit, and the observed frequencies of the outcome align reasonably well with the expected frequencies across the deciles of predicted probabilities. The observed frequencies for the outcome classes (risk of malnutrition and no risk of malnutrition) were compared with the expected frequencies across 10 deciles of predicted probabilities. In groups where the predicted probability was low (groups 1 to 5), the observed number of events (class risk of malnutrition) ranged from 1 to 3, with the majority of cases in the lack of risk of malnutrition class. As the predicted probability increased (groups 6 to 10), the number of observed events (class malnutrition) increased, with group 10 showing 15 observed events against 3 non-events, closely matching the expected values. Thus, the model appears to provide an adequate fit to the data.

#### 3.5.3. Cross-Validation Results

The model achieved an average accuracy = 0.736, indicating that approximately 73.6% of the samples were correctly classified. The Kappa statistic, which adjusts for the possibility of agreement occurring by chance, had a mean value of Kappa = 0.422. This result suggests a moderate level of agreement between the predicted and actual classifications, suggesting that the model performs reasonably well.

The variability in model performance across the cross-validation folds was assessed using the standard deviation of accuracy and Kappa. The standard deviation of accuracy was SDaccuracy = 0.103, and the standard deviation of SDKappa = 0.212, reflecting some degree of variability in the model’s predictive performance across different subsets of the data.

#### 3.5.4. Multicollinearity Testing

The Variance Inflation Factor (VIF) values and tolerance levels suggest that multicollinearity is not a significant issue in this model. The slightly elevated VIF values for ASMI and VAT (both around VIF = 2.3) may indicate some mild collinearity, but they are well below the common threshold of 5, suggesting the model’s estimates are relatively stable and reliable. Therefore, no further action is required to address multicollinearity in this model.

The final model, consisting of seven predictors, explains a significant portion of variability in the outcome, with an R^2^ Tjur = 0.326, based on a sample size of 153 individuals. The results in Table 5 highlight several important predictors of malnutrition risk, as reflected in the odds ratios (ORs) and their respective confidence intervals (CI 95%).

### 3.6. Interpretation of the Regression Coefficient

The odds ratio (OR) of malnutrition risk in older adults who are non-smokers, do not take SSRIs, do not have frailty syndrome, and do not experience a loss of appetite, with median values for appendicular muscle mass index (ASMI: 7.70 kg/m^2^), visceral adipose tissue (1.86 L), and CCK-8 levels (160.6 pg/mL), is 0.12 (equivalent to a probability of 0.11). This indicates a low likelihood of malnutrition under these conditions. The result is statistically significant, with *p* < 0.001, meaning that the model confidently predicts a reduced risk of malnutrition for individuals with this clinical profile.

Smoking was found to significantly increase the odds of malnutrition. Smokers had an OR = 4.54 (95% CI: 1.14–19.08, *p* = 0.033), indicating that smoking increases the odds of malnutrition occurrence by over four times compared to non-smokers.

Similarly, SSRI/MAOI medication intake was also associated with a higher risk of malnutrition, with an OR = 3.01 (95% CI: 0.86–11.55). The association did not reach significance (*p* = 0.092).

Frailty syndrome was a strong and statistically significant predictor of malnutrition. Individuals identified as frail had an OR = 6.60 (95% CI: 2.82–16.24, *p* < 0.001), indicating that those with frailty syndrome are more than six times as likely to experience malnutrition compared to non-frail individuals.

Loss of appetite, as assessed by SNAQ or CNAQ, was also a significant risk factor. Participants reporting loss of appetite had an OR = 2.75 (95% CI: 1.20–6.48, *p* = 0.018), meaning they were nearly three times more likely to develop malnutrition.

However, the Appendicular Skeletal Muscle Mass Index (ASMI), which was centered around a median value of 7.70 kg/m^2^, showed a protective effect. For every 1 kg/m^2^ increase in muscle mass, the OR of malnutrition decreased (OR: 0.61, 95% CI: 0.39–0.88, *p* = 0.015).

The effect of visceral adipose tissue (centered around a median of 1.86 L) was not statistically significant, with an OR = 1.37 (95% CI: 0.90–2.22, *p* = 0.163). Although higher visceral fat may suggest an increased risk of malnutrition, the evidence from this model is inconclusive.

CCK-8 levels, centered around a median of 160.60 pg/mL, were marginally associated with the risk of malnutrition, with an odds ratio of 1.01 (95% CI: 1.00–1.01, *p* = 0.057). This suggests that for every additional pg/mL increase in CCK-8 above 160.60 pg/mL, the odds of malnutrition occurrence increase by approximately 1%.

### 3.7. Adjusted Biomarker Effects in Multivariable Models: Accounting for Covariates and Confounding Factors

The effect of each biomarker (exposure) was modeled using a separate GLM. The exposure effect was further adjusted for covariates that, as presented in Table 1, were significantly associated with the respective exposure or showed a potential confounding effect (*p* < 0.200). Additionally, the fitted models were refined by excluding covariates that demonstrated clear multicollinearity. The results of the adjusted exposure effects are presented in Table A4 in Appendix B.

### 3.8. Evaluating Predictive Accuracy: Discriminatory Performance of the Logistic Regression Model for Malnutrition Risk

**Figure 2:**
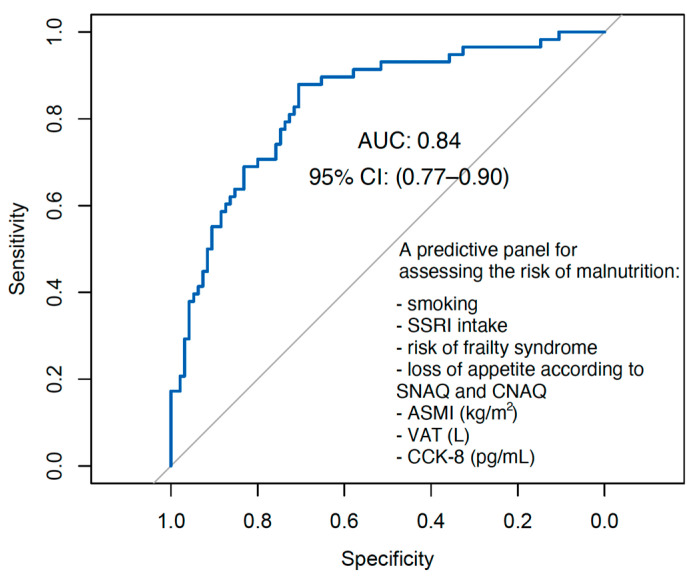
ROC curve for predicting risk of malnutrition in older adults.

An AUC = 0.84 indicates that the logistic regression model has good discriminatory ability in distinguishing between older people who are at risk of malnutrition and those who are not. In clinical terms, this means that the model can correctly rank individuals based on their risk of malnutrition 84% of the time (see visualization by ROC curve in Figure 2).

The 95% confidence interval (0.77–0.90) determined by the DeLong test suggests that, with 95% confidence, the true AUC value lies between 0.77 and 0.90. This provides further evidence of the model’s robustness, as even the lower bound of the confidence interval (0.77) still reflects a model with acceptable to good performance. From a clinical perspective, this suggests that the model is a reliable tool for identifying older adults at higher risk of malnutrition and could be used to inform early intervention strategies such as nutritional assessments or tailored dietary plans.

## 4. Discussion

Malnutrition among older adults represents a significant public health concern due to its multifaceted impact on health outcomes, functional independence, and overall quality of life. With the aging population on the rise, understanding the underlying factors contributing to malnutrition risk is critical for improving diagnostic, preventive, and therapeutic strategies. Traditional assessments often rely on clinical indicators and subjective tools which may not fully capture the complexity of nutritional imbalances in this demographic.

Recent advances in molecular biology and biochemistry have highlighted the potential role of serum biomarkers in identifying individuals at risk of malnutrition. Among these, sirtuin-1 (SIRT-1), cholecystokinin-8 (CCK-8), melatonin, and total antioxidant capacity (TAC) have emerged as possible candidates [125,126,127,128,129].

In this study, these biomarkers were selected because they may reflect metabolic and physiological changes associated with aging and provide possible insights into the interplay between nutrition, oxidative stress, and systemic health.

In the present study, SIRT-1 exhibited a moderate predictive capability in both male and female subjects. While these observations suggest that SIRT-1 has potential as a biomarker for the detection of risk of malnutrition, particularly in settings that prioritize sensitivity, it is essential to note that its utilization should be complemented by additional diagnostic tools to mitigate the occurrence of false positives. As demonstrated by E. Assar et al., SIRT-1 has been identified as a promising biomarker for the assessment of nutritional status in elderly individuals. Their findings indicate that SIRT-1 expression levels exhibit a significant correlation with enhanced nutrition, irrespective of frailty syndrome. Conversely, another study has established a negative correlation between high SIRT-1 levels and frailty syndrome in the elderly, with the association being concurrent with diminished physical performance [130]. In addition, Kumar et al. found that lower levels of SIRT-1 in circulation could serve as a distinctive marker of frailty [131]. The current study offers a more comprehensive understanding of the potential role of SIRT-1 in the regulation of appetite, particularly in the context of the multifaceted mechanisms that lead to malnutrition. The study observed a modest positive correlation between SIRT-1 and loss of appetite. These observations corroborate numerous studies that demonstrated that SIRT-1 regulates the expression of AgRP protein and proopiomelano-cortin neurons, which produce neuropeptides responsible for stimulating appetite (orexigenic effect) and reducing food intake [132,133,134,135].

In contrast, melatonin demonstrated an absence of statistically significant correlations with body composition and muscle strength and weakness measurements. Melatonin’s most notable relationship was a marginally non-significant positive correlation with resting energy expenditure, suggesting that circadian regulation may have a subtle influence on metabolic rate. The paucity of research on this topic is such that the available results are insufficient to reach a single conclusion. As Wolden-Hanson et al. demonstrate in their research, a decline in melatonin secretion with age has the capacity to disrupt energy regulation, resulting in increased body weight, obesity, and associated metabolic consequences [136]. It has been reported that melatonin can affect basal metabolism (BMR) and energy expenditure by regulating metabolism and thermogenesis in rats by acting on brown adipose tissue (BAT), stimulating thermogenesis [137,138,139]. However, the findings are inconsistent and require further study [140].

The present study demonstrated, in an elderly population, a significant positive correlation between TAC and the prevalence of hypertension, cardiovascular disease, hyperlipidemia, and obesity and a negative one with hypothyroidism, which is consistent with other results [141,142,143,144,145,146,147,148,149,150,151,152,153,154,155].

Conversely, considerably higher CCK-8 levels were observed in hyperlipidemic subjects. This finding might be indicative of alterations in appetite regulation and lipid metabolism that are associated with dyslipidemia [156]. This finding suggests that CCK-8 may affect fat metabolism [157]. In the study conducted by Zhou et al., the association between cholecystokinin-induced hyperlipidemia and increased plasma levels of ApoB48 and ApoB100 was investigated. The results of the study suggested that the aforementioned biomarkers could potentially serve as a useful indicator for lipid-related metabolic disturbances [158].

The presented association between TAC, CCK-8, and the prevalence of hypertension, cardiovascular disease, hypothyroidism, hyperlipidemia, and obesity is of significant clinical relevance, as these conditions not only frequently co-occur in the elderly but may also significantly increase the risk of malnutrition in this population, affecting their health status and quality of life [159,160,161,162,163,164].

On the other hand, the findings of this study indicate that elevated antioxidant capacity may be associated with enhanced muscle mass, augmented energy expenditure, and diminished visceral fat. However, the validity of this association remains to be substantiated through further research. The extant literature corroborates the findings of the positive correlation between total antioxidant capacity (TAC) and muscle mass [165,166] and dietary TAC with grip strength [167].

A logistic regression model demonstrated that smoking, the utilization of SSRI/MAOI medication, loss of appetite, the risk of frailty syndrome, decreased ASMI, increased VAT, and increased CCK-8 levels were all significant factors for the risk of malnutrition. Consistent with these findings, other studies have observed that smoking [168,169,170], SSRI/MAOI drug use [117,171], loss of appetite [172], risk of frailty syndrome [173,174,175], and ASMI [176,177] are significant risk factors for malnutrition in older populations. It is important to note that the findings of this study indicated that the risk of frailty syndrome was a strong and statistically significant predictor of risk of malnutrition. Individuals manifesting frailty syndrome exhibited an elevated risk of malnutrition, with a more than sixfold increase in the prevalence of malnutrition compared to those not at risk of frailty syndrome. This finding underscores the significance of incorporating frailty syndrome screening and management strategies within clinical settings to help prevent malnutrition.

In the present study, SIRT-1, melatonin, CCK-8, and TAC were found to have differential diagnostic utility in assessing the risk of malnutrition in elderly individuals. SIRT-1 demonstrated a moderate predictive capability in both sexes (AUC = 0.57). This relatively high sensitivity with low specificity indicates a tendency to generate false positives, which limits its usefulness as a stand-alone biomarker. Assar et al. observed that an elevated expression of the SIRT1 gene is associated with superior nutritional status in elderly individuals regardless of their frailty status [178].

Conversely, melatonin exhibited a notably high sensitivity (women: 0.96; men: 1.00), suggesting its potential for effective detection of malnutrition. However, its extremely low specificity (women: 0.10; men: 0.20) means that almost all well-nourished individuals would be misclassified as being at risk of malnutrition, significantly limiting the clinical use of this biomarker. Despite the existence of studies examining the association of melatonin with other domains, including age-related diseases [179], Alzheimer’s disease [180], osteoporosis prevention [181,182], and sleep efficiency [183], there are no direct studies investigating its role as a biomarker of malnutrition in older individuals.

The findings of the current study revealed that SIRT-1 and melatonin showed no correlation with either disease or smoking, highlighting their potential as independent biomarkers. This is particularly noteworthy as these factors do not influence analyses related to malnutrition risk assessment. Although their predictive value was not established in this study, they may hold potential for future research, especially in individuals with confirmed malnutrition.

This study has identified a novel potential correlation between elevated CCK-8 levels and an increased risk of malnutrition. CCK-8 demonstrated superior performance in comparison to other biomarkers, particularly in the male population, suggesting its potential for identifying malnutrition risk. Furthermore, evidence has shown that older people exhibit higher sensitivity to the appetite-suppressing effects of CCK-8, which may have implications for their nutritional status [184]. Previous research has underscored the role of CCK-8 in the modulation of appetite and its potential impact on nutritional status and aging in the elderly [185,186].

TAC demonstrated a limited diagnostic value, particularly in women, due to its low specificity, resulting in a high number of false positives. In contrast, in male subjects, TAC demonstrated high specificity, thereby effectively excluding cases of malnutrition. However, its low sensitivity constrained its efficacy in identifying risk. Although TAC is frequently used as an indicator of the body’s total antioxidant capacity, compelling evidence to support its effectiveness as a biomarker of malnutrition in older people is lacking.

Given cholecystokinin’s role in appetite regulation [187], elevated levels of CCK-8 have been associated with reduced appetite and food intake in older adults, potentially leading to an increased risk of malnutrition. Studies indicate that aging is linked to higher plasma CCK-8 concentrations, which correlate with decreased appetite and energy intake [188]. Macintosh et al. demonstrated that older adults exhibited higher sensitivity to the appetite-inhibitory effects of CCK-8, resulting in a substantial decrease in food intake. These studies substantiated that the satiating efficacy of CCK-8 was more pronounced in older adults compared to younger adults. Moreover, higher plasma levels of endogenous CCK-8 were observed in older adults both in the fasted state and following a low-calorie meal. Notably, despite elevated CCK-8 concentrations, older adults retained sensitivity to its exogenous effects. This finding suggests that increased endogenous CCK activity may be one of the mechanisms responsible for the anorexia of aging [184]. A meta-analysis by Johnson et al. (2023) demonstrated that the levels of CCK, insulin, leptin, and peptide YY (PYY) are elevated in older individuals. The anorectic effect of these appetite-regulating hormones and the alterations in their levels may represent a pivotal mechanism contributing to the development of anorexia associated with aging [188]. It is evident that appetite disturbance and anorexia are associated with aging. This has been identified as an independent risk factor for frailty, disability, morbidity, and mortality [189,190,191]. The present findings are consistent with the report that higher CCK-8 concentrations may indicate an abnormal satiety response, which in turn could contribute to senile anorexia, especially in malnourished individuals. Reduced CCK receptor function with aging may exacerbate the problem of inadequate food intake [192]. While the present study did not investigate the anorexia of aging, the results regarding CCK-8 may be relevant to the risk of malnutrition in the elderly. The elevated levels of CCK-8 observed in the current study group may suggest its post-natal role in the mechanisms leading to reduced food intake, even in groups that do not manifest the classic symptoms of the anorexia of aging. Further research should focus on determining how differences in CCK-8 levels may contribute to the risk of malnutrition in different health states, with interventions to regulate its action.

The proposed multi-parameter model predicting malnutrition risk confirmed that CCK-8 may play a significant role in the context of malnutrition risk in older people. The model, incorporating smoking, the intake of SSRI/MAOI medications, the risk of frailty syndrome, loss of appetite according to CNAQ and SNAQ, ASMI, VAT, and CCK-8, achieved a high level of accuracy in predicting malnutrition risk, with an AUC of 0.84 (95% CI: 0.77–0.90). From a clinical standpoint, the model can serve as a reliable tool to identify an increased risk of malnutrition in older individuals. It can be utilized to develop early intervention strategies, such as nutritional status assessments or personalized nutrition plans.

### Limitations and Strengths of the Study

This study explores the relationship between SIRT-1, CCK-8, melatonin, and TAC in the context of malnutrition risk among older adults, addressing an area that has received limited attention. By integrating these biomarkers with clinical assessment, this research offers new insights into their potential roles within the mechanisms underlying malnutrition, particularly in the aging population. This integrative approach enhances understanding of the interplay between metabolic, hormonal, and oxidative stress markers and nutritional status, thereby laying the foundation for future investigations in this domain.

However, there are some limitations of the current study. This study did not take into account long-term changes in serum concentrations of the parameters of SIRT-1, CCK-8, melatonin, and TAC. Notably, this study exclusively included individuals at risk of malnutrition, thereby excluding those who were actually malnourished. This may potentially limit the comprehensive understanding of the relationship between the markers analyzed and nutritional status.

Furthermore, caution is essential when interpreting TAC test results, especially in specific clinical contexts, as variations in methodologies, sample handling, and age-related factors may affect the reliability and accuracy of the findings.

## 5. Conclusions

In this study, CCK-8 emerged as a key biomarker among those analyzed, showing relevance in evaluating malnutrition risk in elderly individuals. In contrast, SIRT-1 and melatonin showed limited clinical significance due to their insufficient sensitivity in assessing malnutrition risk. It is important to highlight that the study population consisted of individuals at risk of malnutrition rather than those with confirmed malnutrition. Therefore, future research should investigate the role of SIRT-1 and melatonin in individuals with diagnosed malnutrition.

Among the parameters examined, the utility of TAC appears to be highly context-dependent, as it may hold relevance in conditions characterized by increased oxidative stress or in specific subgroups of older adults. However, its clinical applicability is further constrained by the impact of various diseases, which can influence TAC levels and reduce its reliability as a biomarker for the risk of malnutrition.

The multi-parameter model incorporating CCK-8 provides a more comprehensive assessment of malnutrition risk by integrating key metabolic and hormonal factors. Nevertheless, further studies are required to validate its effectiveness across diverse elderly populations and clinical contexts.

## Figures and Tables

**Table 1:** Distribution of studied parameters for overall sample and with stratification by risk of malnutrition according to MNA.

Characteristic	N	Overall Sample	Risk of Malnutrition		*p* **
			Yes (*n* = 58) *	No (*n* = 95) *	
SIRT-1 (ng/mL)	153	0.92 (0.53, 2.22)	1.11 (0.58, 2.54)	0.84 (0.49, 2.06)	0.265
CCK-8 (pg/mL)	153	160.53 (121.02, 217.24)	175.84 (126.96, 225.41)	156.64 (118.24, 209.97)	0.114
Melatonin (MT) (pg/mL)	153	431.02 (338.12, 494.25)	416.25 (340.14, 471.40)	441.62 (343.43, 499.35)	0.362
TAC (µmol/L)	153	1165.50 (1017.17, 1305.50)	1099.67 (992.17, 1255.92)	1197.17 (1023.00, 1320.50)	0.090

* Mdn (IQR); ** Wilcoxon rank sum test.

**Table 2:** Optimal cut-off points and performance metrics for assayed biomarkers in predicting risk of malnutrition stratified by sex.

Parameter	Sex *	Optimal Cut-Off Point	Accuracy	Sensitivity	Specificity	AUC
SIRT-1	Female	≥0.56 ng/mL	0.54	0.77	0.39	0.57
SIRT-1	Male	≥1.09 ng/mL	0.54	0.80	0.44	0.57
CCK-8	Female	≥168.55 pg/mL	0.61	0.50	0.69	0.58
CCK-8	Male	≥179.44 pg/mL	0.57	0.90	0.44	0.64
Melatonin	Female	≥214.93 pg/mL	0.45	0.96	0.10	0.46
Melatonin	Male	≥382.91 pg/mL	0.43	1.00	0.20	0.51
TAC	Female	≥1028.83 µmol/L	0.47	0.67	0.34	0.42
TAC	Male	≥1682.17 µmol/L	0.77	0.30	0.96	0.54

* (Female *n* = 118; Male *n* = 35).

**Table 3:** Distribution of assayed serum biomarkers stratified by smoking status, hypertension, diabetes, cardiovascular disease, hypothyroidism, hyperlipidemia, and obesity.

Characteristic *n* = 153	SIRT-1 ng/mL	CCK-8, pg/mL	Melatonin, pg/mL	TAC, µmol/L
Smoking status				
Yes (*n* = 14) *	1.43 (0.64, 2.59)	163.35 (124.40, 214.06)	449.64 (357.44, 471.51)	1217.17 (989.67, 1327.17)
No (*n* = 139) *	0.87 (0.51, 2.09)	160.53 (120.18, 216.53)	430.77 (341.96, 497.75)	1165.50 (1020.50, 1298.00)
*p* **	0.308	0.967	0.759	0.960
Hypertension				
Yes (*n* = 83) *	0.67 (0.44, 1.96)	175.85 (137.68, 220.65)	443.17 (380.71, 497.75)	1202.17 (1060.50, 1331.33)
No (*n* = 70) *	1.03 (0.58, 2.62)	151.32 (114.80, 203.44)	416.78 (331.52, 477.79)	1082.17 (952.17, 1263.42)
*p* **	0.119	0.060	0.282	0.004
Diabetes				
Yes (*n* = 22) *	0.77 (0.40, 2.45)	181.52 (149.67, 247.72)	440.90 (328.47, 541.48)	1157.17 (1017.58, 1343.00)
No (*n* = 131) *	0.97 (0.54, 2.13)	158.60 (119.06, 212.82)	431.01 (349.26, 493.73)	1165.50 (1016.33, 1303.00)
*p* **	0.720	0.110	0.956	0.753
Cardiovascular disease				
Yes (*n* = 40) *	0.65 (0.53, 1.78)	181.52 (134.78, 230.37)	433.89 (377.42, 565.13)	1228.00 (1109.25, 1352.58)
No (*n* = 113) *	0.97 (0.53, 2.40)	156.84 (116.19, 204.86)	431.01 (334.51, 484.89)	1102.17 (1007.17, 1278.83)
*p* **	0.416	0.067	0.680	0.030
Hypothyroidism				
Yes (*n* = 26) *	0.66 (0.41, 2.40)	156.74 (108.34, 210.03)	428.42 (382.97, 462.52)	1098.83 (968.00, 1203.00)
No (*n* = 127) *	0.99 (0.55, 2.13)	162.93 (125.01, 217.70)	431.02 (327.95, 499.35)	1183.83 (1020.50, 1325.50)
*p* **	0.503	0.300	0.784	0.047
Hyperlipidemia				
Yes (*n* = 54) *	0.85 (0.45, 2.32)	187.95 (136.00, 230.47)	420.39 (375.94, 495.78)	1170.50 (1026.33, 1318.00)
No (*n* = 99) *	0.97 (0.56, 2.20)	156.72 (117.48, 197.00)	437.73 (332.52, 493.73)	1145.50 (988.83, 1299.67)
*p* **	0.585	0.019	0.756	0.549
Obesity				
Yes (*n* = 69) *	0.88 (0.52, 2.06)	166.26 (129.92, 218.42)	439.56 (367.73, 498.84)	1198.83 (1083.83, 1365.50)
No (*n* = 84) *	0.94 (0.54, 2.42)	157.94 (118.29, 214.58)	425.76 (329.23, 480.62)	1085.50 (953.42, 1255.92)
*p* **	0.822	0.499	0.423	0.005

* Mdn (IQR); ** Wilcoxon rank sum test.

**Table 4:** Correlations of assayed biomarkers with functional and body composition assessments (*n* = 153).

Parameter	SIRT-1		CCK-8		Melatonin		TAC	
	Rho	*p*	Rho	*p*	Rho	*p*	Rho	*p*
GS, kg	0.11	0.194	0.16	0.044	0.13	0.114	0.22	0.006
FS	0.11	0.191	0.02	0.768	−0.08	0.324	−0.13	0.105
RD	0.04	0.649	0.06	0.488	−0.12	0.155	−0.11	0.192
LA	0.14	0.080	0.15	0.059	−0.01	0.916	−0.02	0.790
PS	−0.01	0.222	0.03	0.737	−0.12	0.138	−0.03	0.753
ASMI, kg/m^2^	0.06	0.444	0.12	0.148	0.05	0.564	0.38	<0.001
TBW, L	0.05	0.564	0.13	0.096	0.10	0.221	0.35	<0.001
TEE	0.02	0.778	0.11	0.157	0.14	0.092	0.36	<0.001
REE	0.03	0.702	0.15	0.071	0.14	0.075	0.39	<0.001
FFMI kg/m^2^	0.07	0.413	0.14	0.088	0.03	0.696	0.35	<0.001
FMI kg/m^2^	−0.16	0.054	−0.14	0.077	0.02	0.797	0.09	0.277
PA	0.10	0.235	0.06	0.439	0.2	0.795	0.16	0.055
VAT, L	0.11	0.185	0.22	0.007	0.13	0.115	0.41	<0.001

GS—grip strength; FS—risk of frailty syndrome according to Fried [123]; RD—risk of depression according to GDS; LA—loss of appetite (SNAQ, CNAQ); PS—probable sarcopenia (SARC-F); ASMI—appendicular skeletal muscle mass index; TBW—total body water; TEE—total energy expenditure; REE—resting energy expenditure; FFMI—fat-free mass index; FMI—fat mass index; PA—phase angle; VAT—visceral adipose tissue.

**Table 5:** Regression coefficient of the fitted logistic regression model.

Predictors	Risk of Malnutrition		
	OR	CI 95%	*p*
(Intercept)	0.12	0.05–0.24	<0.001
Smoking			
No	Reference level		
Yes	4.54	1.14–19.08	0.033
SSRI and MAOI medication			
No	Reference level		
Yes	3.01	0.86–11.55	0.092
FS			
No	Reference level		
Yes	6.60	2.82–16.24	<0.001
LA			
No	Reference level		
Yes	2.75	1.20–6.48	0.018
ASMI (centered by Mdn = 7.70 kg/m^2^)	0.61	0.39–0.88	0.015
VAT (centered by Mdn = 1.86 L	1.37	0.90–2.22	0.163
CCK-8 (centered by Mdn = 160.60 pg/mL)	1.01	1.00–1.01	0.057

Notes: SSRI—selective serotonin reuptake inhibitor; MAOI—monoamine oxidase inhibitor; FS—risk of frailty syndrome; LA—loss of appetite according to SNAQ and CNAQ; ASMI—appendicular skeletal muscle mass index; VAT—visceral adipose tissue; CCK-8—cholecystokinin-8.

## Data Availability

The original contributions presented in this study are included in the article. Further inquiries can be directed to the corresponding author.

## Appendix A

**Table A1:** Optimal cut-off points and performance metrics for assayed biomarkers in predicting risk of malnutrition (with risk of malnutrition as positive class).

Parameter	Optimal Cut-Off Point	Accuracy	Sensitivity	Specificity	AUC
SIRT-1	≥0.56 ng/mL	0.50	0.79	0.32	0.55
CCK-8	≥168.55 pg/mL	0.59	0.57	0.61	0.58
Melatonin	≥242.90 pg/mL	0.42	0.95	0.09	0.46
TAC	≥1682.17 µmol/L	0.63	0.05	0.98	0.42

**Table A2:** Correlation coefficients with *p*-values adjusted for multiple comparisons.

Parameter	SIRT-1	CCK-8	TAC
SIRT-1	-	-	Rho = 0.02 *p*_adj_ = 1.000
CCK-8	Rho = 0.12 *p*_adj_ = 0.619	-	Rho = 0.10 *p*_adj_ = 0.873
Melatonin	Rho = 0.18 *p*_adj_ = 0.172	Rho = 0.06 *p*_adj_ = 1.000	Rho = −0.05 *p*_adj_ = 1.000

**Table A3:** Distribution of clinical functional, nutritional, and body composition assessments for overall sample and with stratification by risk of malnutrition.

Characteristic	N	Overall Sample *	Risk of Malnutrition		*p* *
			Yes (*n* = 58) *	No (*n* = 95) *	
GS, kg	153	20.10 (15.50, 25.75)	20.28 (15.40, 25.00)	20.05 (16.13, 28.00)	0.446
FS	153	52.00 (33.99%)	35.00 (60.34%)	17.00 (17.89%)	<0.001 **
RD	153	33.00 (21.57%)	21.00 (36.21%)	12.00 (12.63%)	0.001 **
LA	153	85.00 (55.56%)	45.00 (77.59%)	40.00 (42.11%)	<0.001 **
PS	153	15.00 (9.80%)	11.00 (18.97%)	4.00 (4.21%)	0.003 **
ASMI, kg/m^2^	153	7.66 (6.86, 8.96)	7.27 (6.62, 8.63)	7.85 (7.13, 9.13)	0.025
TBW, L	153	32.63 (29.20, 37.98)	31.11 (28.09, 35.78)	34.00 (29.99, 38.35)	0.050
TEE	153	2204.44 (2028.60, 2410.78)	2173.83 (1996.63, 2308.88)	2223.33 (2045.05, 2446.89)	0.104
REE	153	1348.71 (1255.16, 1480.11)	1297.85 (1241.09, 1442.25)	1364.15 (1286.95, 1488.71)	0.061
FFMI kg/m^2^	153	16.80 (15.45, 18.90)	16.43 (14.96, 18.48)	17.17 (15.65, 19.10)	0.063
FMI kg/m^2^	153	11.00 (8.73, 13.48)	10.87 (8.38, 13.32)	11.24 (8.80, 13.53)	0.443
PA	153	5.31 (5.02, 5.81)	5.15 (4.95, 5.57)	5.48 (5.07, 5.95)	0.010
VAT, L	153	1.86 (1.31, 2.48)	1.78 (1.29, 2.53)	1.89 (1.31, 2.43)	0.683

* overall sample—Mdn (IQR); *n*—(%); *p*—Wilcoxon rank sum test; ** Pearson’s chi-squared test. GS—grip strength; FS—risk of frailty syndrome; RD—risk of depression according to GDS; LA—loss of appetite according to SNAQ and CNAQ; PS—probable sarcopenia; ASMI—appendicular skeletal muscle mass index level; TBW—total body water value; TEE—total energy expenditure; REE—resting energy expenditure; FFMI—fat-free mass index; FMI—fat mass index; PA—phase angle; VAT—visceral adipose tissue.

## Appendix B

**Table A4:** Results of adjusted exposure effects on the risk of malnutrition based on fitted GLM models with logit link function.

Exposure	Malnutrition Risk			N_obs_	R^2^_tjur_
	OR	CI 95%	*p*		
SIRT-1 Centered at Mdn = 0.92 ng/mL	1.03	0.719–1.406	0.857	153	0.265
CCK-8 Centered at Mdn = 160.53 pg/mL	1.01	0.996–1.012	0.065	153	0.190
Melatonin Centered at Mdn = 431.02 pg/mL	1.00	0.998–1.002	0.997	153	0.115
TAC Centered at Mdn = 1165.50 µmol/L	1.00	−0.003–0.010	0.286	153	0.087

Note: OR—odds ratio; CI 95%—95% confidence interval; *p*—*p*-value of the statistical test; N_obs_—number of observations in the fitted model; R^2^_tjur_—Tjur’s coefficient of determination.

The effects of each exposure were adjusted for sex and age. Additionally, the effects were further adjusted as follows:

TAC was adjusted for comorbidities (hypertension, heart diseases, hypothyroidism, obesity), grip strength (centered at the median = 20.1 kg), fat-free mass index (FFMI; centered at the median = 16.8 kg/m^2^), and phase angle (centered at the median = 5.31).

SIRT-1 was adjusted for comorbidity occurrence (hypertension), grip strength (centered at the median = 20.1 kg), risk of frailty syndrome, loss of appetite (measured by SNAQ and CNAQ), fat mass index (FMI; centered at the median = 11.0 kg/m^2^), and visceral adipose tissue (VAT) (centered at the median = 1.86 L).

CCK-8 was adjusted for comorbidities (hypertension, diabetes mellitus, heart diseases, hyperlipidemia), grip strength (centered at the median = 20.1 kg), loss of appetite (measured by SNAQ and CNAQ), total energy expenditure (centered at the median = 2204.44 kcal), FFMI (centered at the median = 16.8 kg/m^2^), FMI (centered at the median = 11.0 kg/m^2^), and visceral adipose tissue (centered at the median = 1.86 L).
